# Use of Telemedicine for Chronic Liver Disease at a Single Care Center During the COVID-19 Pandemic: Prospective Observational Study

**DOI:** 10.2196/20874

**Published:** 2020-09-21

**Authors:** Maria Guarino, Valentina Cossiga, Andrea Fiorentino, Giuseppina Pontillo, Filomena Morisco

**Affiliations:** 1 Gastroenterology Unit Department of Clinical Medicine and Surgery University of Naples Federico II Naples Italy

**Keywords:** telemedicine, COVID-19, hepatology, telehealth, liver disease, Italy, hospital, chronic disease, liver

## Abstract

**Background:**

The COVID-19 outbreak has overwhelmed and altered health care systems worldwide, with a substantial impact on patients with chronic diseases. The response strategy has involved implementing measures like social distancing, and care delivery modalities like telemedicine have been promoted to reduce the risk of transmission.

**Objective:**

The aim of this study was to analyze the benefits of using telemedicine services for patients with chronic liver disease (CLD) at a tertiary care center in Italy during the COVID-19–mandated lockdown.

**Methods:**

From March 9 to May 3, 2020, a prospective observational study was conducted in the Liver Unit of the University Hospital of Naples Federico II to evaluate the impact of (1) a fully implemented telemedicine program, partially restructured in response to COVID-19 to include video consultations; (2) extended hours of operation for helpline services; and (3) smart-working from home to facilitate follow-up visits for patients with CLD while adhering to social distancing regulations.

**Results:**

During the lockdown in Italy, almost 400 visits were conducted using telemedicine; only patients requiring urgent care were admitted to a non–COVID-19 ward of our hospital. Telemedicine services were implemented not only for follow-up visits but also to screen patients prior to hospital admission and to provide urgent evaluations during complications. Of the nearly 1700 patients with CLD who attended a follow-up visit at our Liver Unit, none contracted COVID-19, and there was no need to alter treatment schedules.

**Conclusions:**

Telemedicine was a useful tool for following up patients with CLD and for reducing the impact of the COVID-19 pandemic. This system of health care delivery was appreciated by patients since it gave them the opportunity to be in contact with physicians while respecting social distancing rules.

## Introduction

COVID-19, caused by the recently identified coronavirus SARS-CoV-2, has been spreading rapidly across the world since December 2019. The COVID-19 pandemic constitutes a great health emergency, with 3,090,445 confirmed cases and 217,769 deaths globally, as of April 30, 2020 [1]. Most patients experienced fever, dry cough, and asthenia, but there is a wide spectrum of minor symptoms, such as diarrhea, anosmia, and ageusia. Patients experiencing a severe course of disease had a higher incidence of pneumonia and acute respiratory distress syndrome, and required oxygen and mechanical ventilation more often, compared to those with nonsevere disease [2].

Patients with comorbidities, such as hypertension, diabetes, and obesity, as well as older adults, have a higher risk of complications and mortality [3]. There is a lack of data on the course of COVID-19 in subjects with pre-existing liver diseases, but it is reasonable to conclude that this category of patients are also at a higher risk of severe outcomes. For example, patients with autoimmune liver disease or liver cancer undergoing immunosuppressive therapy, or cirrhotic patients with an altered immune response would be more susceptible to infections [4].

To contain the rapid spread of COVID-19, social distancing became a necessary prevention strategy [5]. However, pandemic responses are disrupting routine care for non–COVID-19 patients. It has become fundamental to define the prioritization criteria for outpatient visits, to avoid unnecessary in-hospital visits, and to facilitate the management of patients in the home setting. Telemedicine, a virtual care platform that allows communication between health care professionals and patients, has been proposed as an indispensable tool to reduce COVID-19 spread and to maintain care of patients with chronic disease [6-8]. The World Health Organization [9] described telemedicine as:

The delivery of health care services, where distance is a critical factor, by all health care professionals using information and communication technologies for the exchange of valid information for diagnosis, treatment and prevention of disease and injuries, research and evaluation, and for the continuing education of health care providers, all in the interests of advancing the health of individuals and their communities.

Telemedicine is now well accepted because it is patient centered, protects patients and clinicians from viral exposure, and provides health outcomes comparable to traditional methods of health care delivery without compromising the patient-clinician relationship and enhancing patient satisfaction [10].

In particular, subjects with chronic disease, such as chronic liver disease (CLD), require assistance from their physician via scheduled outpatient appointments to solve daily clinical issues (ie, monitoring of patient weight, diuresis, and serum electrolytes during a diuretic treatment for decompensated cirrhosis, or checking heart rate in patients undergoing nonselective β-blockers treatment for the first time for variceal bleeding prevention). For these reasons, a telemedicine service has been in effect since 2015 at the Liver Unit of the University Hospital of Naples Federico II. In 2020, it was already fully implemented with well-trained physicians. The only equipment requirements necessary for this service were smartphones, printers, laptops, and tablet computers with an internet connection. This service allows us to stay in contact with patients daily by email, fax, and phone call. During the last 5 years, we improved the quality of clinical care, achieving patient acceptability and trust. Moreover, the use of text messaging, calling, and remote monitoring decreased the number of unscheduled visits. During the COVID-19 outbreak, we further improved telemedicine services to follow international recommendations [11-14] and postpone nonurgent outpatient visits.

The aim of this study was to prospectively analyze the benefits of using telemedicine for patients with CLD in a tertiary care center during the COVID-19 lockdown in Italy.

## Methods

A prospective observational study was conducted to evaluate a telemedicine service set up for patients with CLD from March 9 to May 3, 2020, at the Liver Unit of the University Hospital of Naples Federico II during the lockdown. During this period of social distancing, high-acuity care was prioritized, and elective procedures and routine care were postponed.

In the last 2 months, several hospitals in Italy and their liver units became COVID-19 hotspots and designated wards during the pandemic, and patients with CLD were instructed to remain at home to avoid the risk of infection. In the Campania region, there were fewer cases than in regions of Northern Italy (4331 confirmed cases, as of April 25, 2020) [15]. For this reason, our Liver Unit continued to take care of patients with CLD but changed standard procedures for outpatient care. The waiting room, for example, was readapted to facilitate adequate distancing between patients requiring necessary visits, waiting time were reduced, and companions were not permitted.

The telemedicine service, implemented for patients with CLD in 2015, was organized as follows:

- Helpline: available from Monday to Friday between 2 PM and 5 PM, managed by 5 trained physicians. This service collects all requests for medical consultations, concerns with treatment plans, side effects of drugs, visit schedules, and state of disease. This real-time modality allows specialists, general practitioners, and patients to speak with one another in real time to discuss conditions;
- Secured email and fax services: available 24 hours a day, aimed to send treatment schedules, laboratory tests results, medical imaging such as X-rays, photos, ultrasound recordings, or other static and video medical imaging to remote specialists for analysis and future consultation, which is particularly relevant for patients with CLD requiring close monitoring.

During the lockdown in Italy, these services were further improved to limit face-to-face contact by implementing the following:

- Video consultations (through Skype or WhatsApp). In the beginning, during Italy’s state of emergency, Skype and WhatsApp were used for video consultations due to the lack of a secure telemedicine software, which was made available in our Liver Unit starting in May 2020;
- Extended helpline hours, now available from Monday to Friday between 8 AM to 1 PM and 3 PM to 6 PM, as well as during weekends;
- Smart-working from home to increase remote patient monitoring and mobile health care while reducing the risk due to contact with medical staff.

## Results

**Figure 1:**
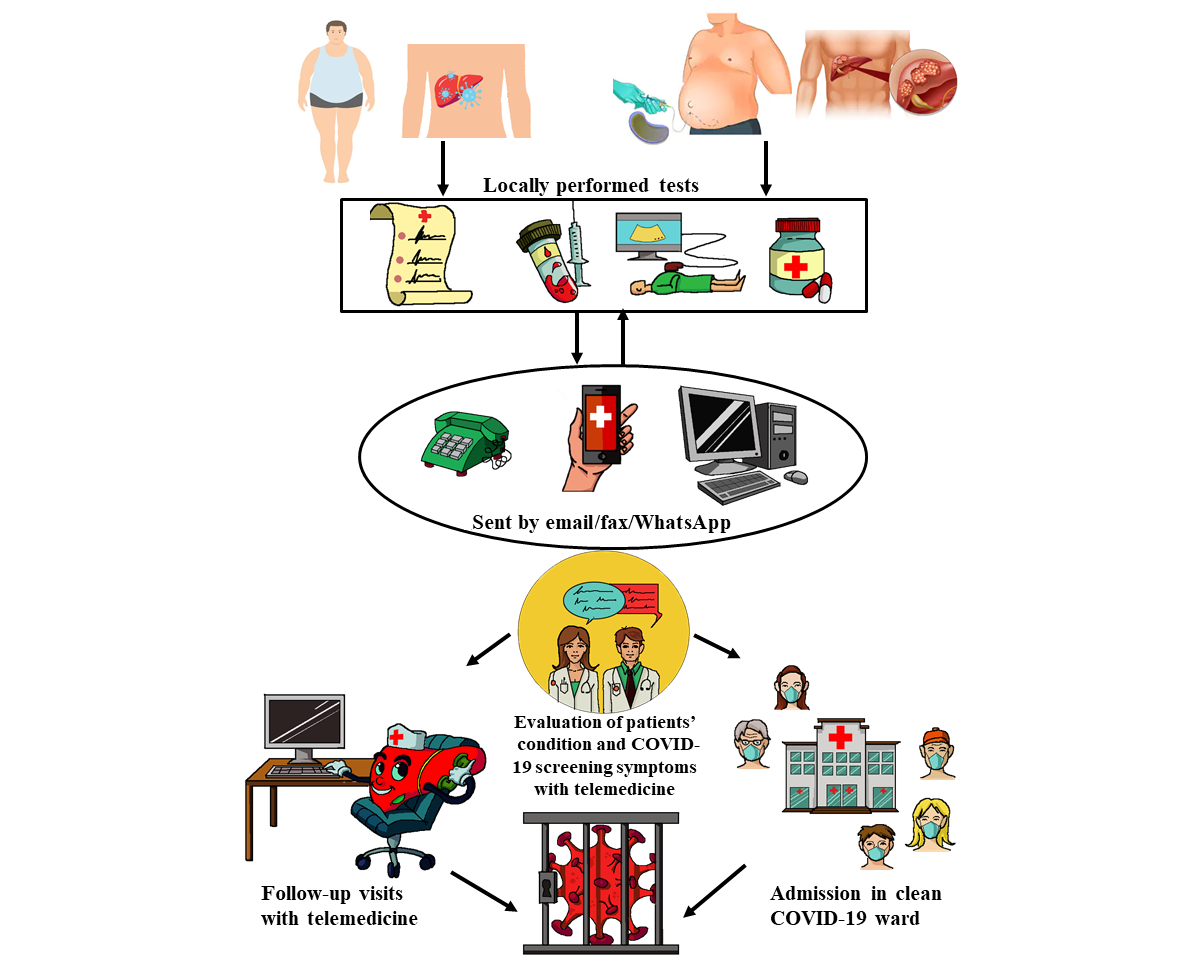
Graphical representation of telemedicine activity during the COVID-19 pandemic.

During Italy’s lockdown, 480 outpatient visits were scheduled but, in order to avoid hospital admissions, most were conducted via telemedicine (Figure 1). In-hospital visits were limited only to patients with urgent needs or with oncological conditions, since international regulations on social isolation and guidelines from major hepatology associations encouraged conducting appointments via telemedicine [11-14].

In particular, 80 follow-up visits (in a population of 250 patients) were scheduled for patients with chronic viral hepatitis (hepatitis B virus or hepatitis C virus) undergoing antiviral treatment (nucleos(t)ide analogues or direct-acting antivirals). For these patients, clinical conditions were evaluated by phone and laboratory tests; ultrasound was performed locally and follow-up prescriptions were sent by secure email and/or fax. Similarly, 120 follow-up visits (in a population of 1000 patients) were scheduled for patients who had obtained a sustained virological response after antiviral treatment (interferon or direct-acting antiviral schedules); for these patients, clinical conditions were evaluated by teleconsultations and laboratory tests; ultrasound was performed locally and follow-up prescriptions were sent by email and/or fax.

Subsequently, 40 outpatient visits in a population of 200 patients with alcoholic liver disease, nonalcoholic fatty liver disease, or nonalcoholic steatohepatitis were replaced by teleconsultations and full telemedicine services. In addition, other conditions, such as primary biliary cholangitis, primary sclerosing cholangitis, and autoimmune hepatitis were managed in the same way (approximately 30 outpatient visits in a population of 60 patients). At our center, immunosuppressive treatment for autoimmune hepatitis was not modified in any way.

Additionally, 15 outpatient visits in a population of 40 patients with CLD associated with a rare disease (eg, cystic fibrosis, common variable immunodeficiency disorders, Crigler-Najjar syndrome, hereditary hemochromatosis, Alagille syndrome, congestive hepatopathy, nodular regenerative hyperplasia, etc) were managed with telemedicine services.

Patients with decompensated cirrhosis are at risk of worse outcomes if infected with COVID-19; hence, it is necessary to consider minimizing these patients’ exposure to medical staff. During the lockdown period, we managed 50 patients with compensated cirrhosis by teleconsultations, stressing the importance of prophylaxis measures for hepatic encephalopathy and spontaneous bacterial peritonitis to avoid decompensation and reduce hospital admissions. Laboratory tests were performed locally to evaluate electrolytes and renal function in patients with ascites undergoing diuretic therapy. In patients requiring evacuative paracentesis, this procedure was conducted aseptically in a non–COVID-19 ward of the hospital (15 admissions).

Thirty outpatient visits for patients with a previous diagnosis of hepatocellular carcinoma (HCC), who were “disease-free” at the time, were replaced by telemedicine. Conversely, patients with HCC (a new diagnosis or disease recurrence) were managed as usual in a non–COVID-19 ward of the hospital (30 admissions). In particular, HCC staging and treatment were provided; we were able to offer patients several options according to HCC burden and stage (surgery, ablation techniques, transarterial chemoembolization / transarterial embolization, and systemic treatments). Moreover, for patients with HCC requiring follow-ups to evaluate treatment response, imaging tests were unaffected by the lockdown and were conducted in a timely manner. For HCC screening (performed in ultrasound centers in the Campania Region), ultrasounds was delayed, since according to International Liver Cancer Association guidelines, delays of 1-2 months in HCC screening do not significantly increase risk [13].

Among the approximately 70 liver transplanted patients handled at our center, 50 follow-up visits were scheduled during the lockdown, but the majority was managed via telemedicine. Laboratory tests, including assessment of immunosuppressive drug levels, were performed locally. Only 15 patients had clinical conditions requiring in-hospital visits and close face-to-face monitoring (due to cytomegalovirus infection, immunosuppressive drugs toxicity management, biliary complications, management of comorbidities exacerbation like Crohn disease, and phlebotomy in patients with polyglobulia). Even among transplant patients, immunosuppressive treatment was not modified.

Overall, 200-250 tele- or video consultations (over the course of 9 weeks) were conducted per week and 130-150 phone calls were received, while 100-150 emails and/or faxes were received per week and 150-200 were sent, replacing outpatient visits. Patients expressed satisfaction with this method of management and did not encounter any difficulties with contacting us, since they were strongly aware of the risks associated with leaving home.

In addition to helping patients to manage chronic liver diseases, the primary goals of implementing a telemedicine system were as follows:

1. Screen patients for COVID-19 prior to admission (inpatients; outpatient visits for HCC or decompensated chronic advanced liver disease or liver transplant patients) according to the guidelines [16] implemented to keep our center safe;
2. Provide telemedicine follow-up visits for all patients with CLD and nonurgent conditions for a face-to-face visit;
3. Provide routine care to our patients with CLD, including promptly addressing questions, coordinating complex care, offering caregiver support (eg, in case of hepatic encephalopathy through early recognition and initiation of prophylactic therapies), nutritional advices, and early interventions to ensure drugs compliance (especially for immunosuppression drugs in liver transplant patients), and to prevent decompensation for chronic advanced liver disease while enabling patients to stay at home;
4. Provide urgent evaluations during decompensation events to minimize emergency room visits and admissions.

At the end of this period, none of nearly 1700 patients with CLD who followed up at our Liver Unit contracted COVID-19. This success was possibly due to measures adopted by the Italian government and the medical care guaranteed by our established and further improved telemedicine services.

## Discussion

Telemedicine is a rapidly expanding health care delivery modality with increasing utility for health care. During the COVID-19 pandemic, telemedicine became a useful tool for remotely connecting patients with health care providers and facilitating follow-up visits via smartphones or webcam-enabled computers.

In the management of patients with CLD, telemedicine has already been successful in different cases, such as hepatitis C therapy in prison populations or to enhance the efficiency of liver transplant evaluations [17-19]. The current study has shown that a fully implemented telemedicine service, partially restructured for the COVID-19 pandemic, can convert more than 75% of planned outpatients visits to remote appointments.

Moreover, the impact of the COVID-19 pandemic on routine health care should not be underestimated, since it has resulted in the need to completely change usual follow-up procedures, such as frequent patient-physician contact to evaluate disease status, screen for complications, and assess response to therapy. Tapper and Asrani [20] described the ways in which COVID-19 impacted the quality of cirrhosis care and discussed this impact in three waves: (1) a delay in routine care for cirrhotic patients such as deferred screening for HCC and for varices with late diagnosis, or canceled elective therapeutic procedures; (2) a backlog of outpatient visits, resulting in a large workload for physicians and an increase in acute decompensation in patients classified as low risk in the first wave when normal clinical care is resumed; (3) the consequences due to missed diagnosis that will persist for years to come. The authors identified several measures to avoid these complications like the use of telemedicine as a substitute for outpatient visits during social distancing [20].

Our study showed that a sizeable proportion (about 75%) of outpatient visits in a CLD setting can be managed effectively with telemedicine without compromising patients’ health or quality of care. Furthermore, with our telemedicine system we did not postpone appointments, preventing any unintended loss to follow-up, since we scheduled a new appointment for each patient according to their specific condition. Above all, we managed to avoid a surge in outpatient activity after the lockdown due to a backlog of postponed care.

No resistance was encountered by patients to using this modality of care delivery since it was both familiar to them and kept both patients and medical staff protected against COVID-19. Indeed, the prevalence of high-speed internet and smartphones makes it possible to apply this framework easily to set up video teleconsultations from a patient’s home. Patients highlighted the faster and increased availability of medical care through telemedicine. Moreover, they were thankful to receive an expert opinion whether it was a reassurance or a clinical evaluation.

After the lockdown, we plan to continue with telemedicine services to keep in contact and manage in a better way patients who cannot come to the hospital for scheduled visits due to work-related obligations or disabling pathologies. Moreover, telemedicine could be used in the future to follow up with specific categories of patients, such as hepatitis C long-term responders, to prevent in-hospital overcrowding.

It should be acknowledged that telemedicine services have some limitations: (1) the absence of physical interactions (eg, eye contact, handshake), which may play a comforting role for patients; (2) interferences, lapses, delays, or interruptions due to service connection problems; and (3) difficulties associated with communicating bad news [21]. In a time of crisis, such as the COVID-19 pandemic, these difficulties can be overcome but must be taken into considerations if the use of telemedicine is to be integrated into routine clinical care. In fact, telemedicine needs to be defined by national regulations and frameworks for public health emergencies. A plan is necessary to increase physicians’ expertise and monitor patients remotely, as well as to educate the population on the correct use of this service before it can be globally adopted [8].

The COVID-19 pandemic has dramatically changed several aspects of health care; during the lockdown, it became essential to use telemedicine and virtual software. In Italy, there are no national regulations for telemedicine use; only in some tertiary care centers were there remote services such as secured mail, fax, and dedicated helplines to follow up with patients. During COVID-19 outbreak, each Italian region, including Campania, has attempted to implement remote assistance systems to manage patients with chronic disease.

One limitation of our study is its cross-sectional design. A longitudinal study evaluating the long-term impact of telemedicine on the course of CLD would provide additional data to improve the system and to optimize outcomes for remote patients in order to reduce missed diagnoses, progressive disease outcomes, and loss to follow-up. In this context, additional studies are needed to evaluate the use of telemedicine in the long term to enhance, and not replace, the current standard of care.

In conclusion, the COVID-19 pandemic has deeply strained health care systems around the world. To mitigate the impact of disease, it is fundamental to minimize the risk of patients’ and physicians’ exposure to the virus; in this scenario, telemedicine played an important role. Although telemedicine will not solve all health-related problems, its well-standardized use in our Liver Unit demonstrated its utility in times of social distancing. Finally, the use of telemedicine systems provided us with the opportunity to conduct scheduled visits remotely for patients with CLD while reducing a postlockdown surge in outpatient visits.

## Abbreviations

CLD: chronic liver disease
HCC: hepatocellular carcinoma
